# Acute kidney injury in SARS-CoV-2 infected patients

**DOI:** 10.1186/s13054-020-02872-z

**Published:** 2020-04-16

**Authors:** Vito Fanelli, Marco Fiorentino, Vincenzo Cantaluppi, Loreto Gesualdo, Giovanni Stallone, Claudio Ronco, Giuseppe Castellano

**Affiliations:** 1 Department of Surgical Science, Division of Anesthesia and Critical Care Medicine, University of Turin, AOU Città della Salute e della Scienza di Torino, Turin, Italy; 2 Nephrology Unit, “Mons. Dimiccoli” Hospital Barletta, Barletta, Italy; 3 Nephrology and Kidney Transplantation Unit, Department of Translational Medicine and Center for Autoimmune and Allergic Diseases (CAAD), University of Piemonte Orientale (UPO), “Maggiore della Carità” University Hospital, Novara, Italy; 4 Nephrology, Dialysis and Transplantation Unit, Department of Emergency and Organ Transplantation, University of Bari, Bari, Italy; 5 Nephrology, Dialysis and Transplantation Unit, Department of Medical and Surgical Science, University of Foggia, Foggia, Italy; 6 Department of Nephrology Dialysis and Transplantation, International Renal Research Institute of Vicenza, San Bortolo Hospital, Vicenza, Italy

## Background

Severe acute respiratory syndrome coronavirus-2 (SARS-CoV-2) is the pathogen responsible of atypical pneumonia that has affected more than 330,000 people and caused death of more than 14,000 patients (as of March 23, 2020, WHO Report). Coronavirus spike (S) glycoproteins mediate entry into cells end predominantly lung epithelial alveolar cells precipitating an interstitial pneumonia that evolves through acute respiratory distress syndrome (ARDS) [1]. Interestingly, about 25% of acute kidney injury (AKI) occurrence has been reported in this clinical setting [2, 3]. These data were recently confirmed by the Italian Report of “Istituto Superiore di Sanità” describing an incidence of 27.8% in more than 2000 patients (updated on 17 March) (https://www.epicentro.iss.it/coronavirus/bollettino/Report-COVID-2019_17_marzo-v2.pdf). In this commentary, we discuss possible mechanisms of the COVID-19-induced AKI, indicating potential new approaches for risk stratification of SARS-CoV-2 infected patients that may help clinical decision in this emerging scenario.

## Main text

AKI represents a life-threatening complication in critically ill patients, often leading to increased risk of death. As recently reported by Wilson et al., the onset of moderate-to-severe AKI described a higher-risk subset of ARDS patients, with a significant risk for mortality [4]. Considering the possible pathogenic mechanisms of AKI-associated ARDS, AKI may be ascribed to different causes such as impairment of gas exchange, hemodynamic alterations including right heart failure, fluid overload and systemic congestion, injurious mechanic ventilation strategies, and development of secondary infections/sepsis. Several studies emphasized the relevance of the inflammatory/immune-mediated reaction with the release of high levels of circulating harmful mediators capable to interact with kidney-resident cells causing endothelial dysfunction, microcirculatory derangement, and tubular injury [5]. In accordance with previous studies, the beta coronaviruses SARS-CoV and the most recent SARS-CoV-2 use angiotensin-converting enzyme 2 (ACE-2) as receptor to facilitate viral entry into target cells; ACE-2 is also located on the surface of kidney tubular cells, and their infection may worsen the local inflammatory response and consequently the incidence and the duration of AKI episodes [6] (Table 1).

**Table 1:** Key points of acute kidney injury in SARS-CoV-2 infected patients

- AKI is frequently observed in ARDS patients affected by different comorbidities: similar findings were observed in Wuhan COVID-19 infected patients.	
- ARDS-associated AKI may be ascribed to several causes including an inflammatory/immune reaction characterized by an enhanced release of circulating mediators able to interact and damage kidney-resident cells.	
- Kidney epithelial cell viral infection may worsen local inflammatory response and consequently the incidence and the duration of AKI episodes.	
- These comorbidities may be associated with a pre-existing chronic decline of kidney function (concept of renal functional reserve) and with a tendency to develop AKI episodes.	
- Identification of patients with AKI may lead to a better allocation of hospital resources: early biomarkers of AKI may favor this process.	
- The use of extracorporeal blood purification techniques and anti-viral therapies may theoretically limit the systemic and local inflammatory response at least in part responsible for multiple organ failures including AKI.	

As described, AKI developed in average 9 days after admission together with secondary infections and acute cardiac damage [2, 3]. Age, severity of illness, and the presence of diabetes are risk factors for AKI in ARDS patients; moreover, the severity of AKI is further associated with BMI and history of heart failure also defined as cardio-renal syndrome [7]. Similar observations, including the presence of diabetes, have been reported for COVID-19-associated ARDS. All these risk factors added to the increased incidence of AKI in elderly lead to the hypothesis that renal complications are predominant in patients with pre-existing chronic impairment of kidney function that is difficult to evaluate based only on serum creatinine levels, thus claiming for the use of new biomarkers of early kidney injury.

Therefore, determining the risk for developing AKI in SARS-CoV-2 infected patients or progressing to severe AKI requiring renal replacement therapies [8] is an important step for the patient’s prognosis and for early implementation of preventative and protective measures [7, 9]. Classical assessment of AKI is still based on serum creatinine and urine output, but they represent only indicators of established kidney damage. In this scenario, much attention has focused on novel biomarkers in the last years, particularly on markers of acute tubular stress/damage such as TIMP-2 (tissue inhibitor of metalloproteinase 2) and IGFBP7 (insulin-like growth factor binding protein 7) and their product [TIMP-2]*[IGFBP-7] identified using the NephroCheck Test [10]. This test has been set for the prediction of moderate to severe AKI within 12 h after ICU admission, and it is the only one approved by the Food and Drug Administration in this setting [11]. Several evidences suggested that the application of NephroCheck may help physicians to identify patients with tubular stress, before kidney dysfunction is manifested [12]. Critically ill patients are indeed exposed to several potential kidney insults in this setting (SARS-CoV-2 infection, drug nephrotoxicity, contrast media) during ICU stay; serial measurements of these biomarkers may be a useful tool to predict AKI during the first 7 days of ICU stay [13].

A positive test suggesting a high risk of developing AKI may require an early nephrology consultation [7], the close monitoring of creatinine and urine output, the optimization of volume and hemodynamic status, the avoidance of iodinated contrast procedures when possible and the use of nephrotoxic drugs (e.g., aminoglycosides, ACE inhibitors, NSAIDs), or the close monitoring of drug levels (vancomycin) [7–14].

## Conclusions

ARDS patients developing AKI are a higher risk for severe outcomes and mortality. In our opinion, it is compelling to establish a prediction model to stratify SARS-CoV-2 infected patients according to AKI severity; this approach might lead to an innovative biomarker-based interventional strategy in this emerging clinical contest leading to a better allocation of hospital resources.

## Data Availability

Not applicable
